# Automated Radiotherapy Planning for Patient-Specific Exploration of the Trade-Off Between Tumor Dose Coverage and Predicted Radiation-Induced Toxicity—A Proof of Principle Study for Prostate Cancer

**DOI:** 10.3389/fonc.2020.00943

**Published:** 2020-06-30

**Authors:** Rik Bijman, Linda Rossi, Abdul Wahab Sharfo, Wilma Heemsbergen, Luca Incrocci, Sebastiaan Breedveld, Ben Heijmen

**Affiliations:** Department of Radiation Oncology, Erasmus MC Cancer Institute, Rotterdam, Netherlands

**Keywords:** personalized radiotherapy, automated multi-criterial treatment planning, normal tissue complication probability (NTCP), prostate cancer, gastro-intestinal

## Abstract

**Background:** Currently, radiation-oncologists generally evaluate a single treatment plan for each patient that is possibly adapted by the planner prior to final approval. There is no systematic exploration of patient-specific trade-offs between planning aims, using a set of treatment plans with a-priori defined (slightly) different balances. To this purpose, we developed an automated workflow and explored its use for prostate cancer.

**Materials and Methods:** For each of the 50 study patients, seven plans were generated, including the so-called clinical plan, with currently clinically desired ≥99% dose coverage for the low-dose planning target volume (PTV_Low_). The six other plans were generated with different, reduced levels of PTV_Low_ coverage, aiming at reductions in rectum dose and consequently in predicted grade≥2 late gastro-intestinal (GI) normal tissue complication probabilities (NTCPs), while keeping other dosimetric differences small. The applied NTCP model included diabetes as a non-dosimetric predictor. All plans were generated with a clinically applied, in-house developed algorithm for automated multi-criterial plan generation.

**Results:** With diabetes, the average NTCP reduced from 24.9 ± 4.5% for ≥99% PTV_Low_ coverage to 17.3 ± 2.6% for 90%, approaching the NTCP (15.4 ± 3.0%) without diabetes and full PTV_Low_ coverage. Apart from intended differences in PTV_Low_ coverage and rectum dose, other differences between the clinical plan and the six alternatives were indeed minor. Obtained NTCP reductions were highly patient-specific (ranging from 14.4 to 0.1%), depending on patient anatomy. Even for patients with equal NTCPs in the clinical plan, large differences were found in NTCP reductions.

**Conclusions:** A clinically feasible workflow has been proposed for systematic exploration of patient-specific trade-offs between various treatment aims. For each patient, automated planning is used to generate a limited set of treatment plans with well-defined variations in the balances between the aims. For prostate cancer, trade-offs between PTV_Low_ coverage and predicted GI NTCP were explored. With relatively small coverage reductions, significant NTCP reductions could be obtained, strongly depending on patient anatomy. Coverage reductions could also make up for enhanced NTCPs related to diabetes as co-morbidity, again dependent on the patient. The proposed system can play an important role in further personalization of patient care.

## Introduction

The aim of radiotherapy treatment planning is to define a treatment that provides adequate tumor volume irradiation with the highest expected therapeutic ratio. To this purpose, doses in organs at risk (OARs) are minimized based on known risks for radiation-induced toxicity (1). Technical developments in external beam radiotherapy (EBRT), e.g., replacement of 3D-conformal radiotherapy (3DCRT) by intensity modulated radiation therapy (IMRT) and volumetric modulated arc therapy (VMAT) (2–4), and improvements in image guidance (5–7), have significantly improved treatment outcome and/or reduced radiation induced side effects in a variety of treatment sites. Recently, developments in automation of treatment planning have further enhanced opportunities for generation of high quality treatment plans (8–10).

Ideally, toxicity risks to be used in planning are modeled with normal tissue complication probabilities (NTCPs). There is an active field of research developing these predictive models (1, 11–15). More and more, published NTCP models include non-dosimetric parameters that modulate the radiation-induced toxicity risk (16). For example, Cozzarini et al. (14) used multivariate logistic regression to include both dosimetric parameters, extracted from the clinical plans, and patient characteristics (e.g., smoking status, age, application and duration of hormonal therapy) in the toxicity prediction models. Pre-selection of a relevant predictor subset was performed using univariate logistic regression. A similar approach was performed in previous work by Sharfo et al. (17) who developed a multivariate logistic regression model predicting radiation induced gastro intestinal (GI) toxicity.

Current practice in radiation therapy treatment planning is based on treatment site specific clinical protocols, containing hard constraints, and planning aims. Evidence based medicine recommends the definition of clinical protocols, based on findings in prospective clinical trials and dose escalation studies (18). Generally, the planning protocol is used by a planner to generate for each patient a single treatment plan that may or may not be adjusted after discussion with the treating physician prior to final approval. There is no systematic exploration of patient-specific trade-offs between the various planning aims by generation of a set of treatment plans for each patient with (slightly) different trade-offs.

We hypothesized that generation of a limited set of well-designed treatment plans per patient, instead of a single plan, can help to better identify plans with optimal patient-specific trade-offs. For example, for some patients with specific anatomies, a slight decrease in coverage might result in a relatively large NTCP gain. For patients with non-dosimetric conditions that result in a significantly enhanced predicted NTCP, a lower PTV coverage or a somewhat enhanced NTCP for a different side-effect might be accepted to counter-act the enhancement. We also hypothesized that automated planning can be used to effectively generate the required treatment plans.

In this paper we have investigated these hypotheses for treatment of prostate cancer. An automated planning algorithm was used to generate for each patient a set of plans to explore the trade-off between the dose coverage of the large planning target volume to be irradiated with reduced dose (PTV_Low_) and the predicted NTCP for grade ≥2 GI toxicity for otherwise similar dose distributions. In particular, measures were taken to maintain clinical target volume (CTV) coverage at 100% and to keep the coverage of the (smaller) PTV_High_ at the requested ≥99% level. Deterioration of bladder dose was also to be avoided. We also investigated to what extent reduction in PTV_Low_ coverage could compensate for significantly enhanced toxicity risks caused by diabetes.

## Materials and Methods

### Patients and Clinical Protocol

Fifty arbitrarily selected prostate cancer patients, previously treated in our center in the context of the randomized HYPRO trial (19) with a simultaneously integrated boost technique, were included in the study. PTV_High_ consisted of the prostate (CTV_High_) expanded with a 5–6 mm isotropic margin, but avoiding overlap with the rectum. PTV_Low_ was defined by applying a 8–10 mm isotropic margin around the prostate + seminal vesicles (CTV_Low_). All patients were treated in the hypofractionation arm with prescribed total doses for PTV_High_ and PTV_Low_ of 64.6 Gy and 57.76 Gy, delivered in 19 fractions. For both PTVs, the planning aim was to have ≥99% of the volume covered by 95% of the prescription dose, with full coverage of the CTVs. Contoured organs at risks (OARs) were rectum, bladder, anus, and hips. Reduction of rectum dose was the highest OAR priority.

### System for Automated Plan Generation

In this study, all treatment plans were generated with the in-house developed Erasmus-iCycle system for fully-automated multi-criterial plan generation, which has been extensively described in the literature (8, 20–22). Generated plans are Pareto-optimal and often superior to manually generated plans (10, 23, 24). Here a short description of the system provided. Plans are generated using a so-called wish-list (described in more detail in section Wish-Lists) that defines the protocol for automated plan generation, based on a set of cost functions that are either defined as hard constraints or planning objectives with assigned priorities and goal values. In plan generation, planning constraints are never violated. On the other hand, goal values of objective functions are met as well as possible or possibly superseded, taking into account the constraints and ascribed priorities. Planning objectives are sequentially optimized according to their priorities while always adhering to all imposed constraints. After each objective function optimization, a new constraint is added to the optimization problem to ensure that the previously obtained function value is maintained while minimizing lower priority objectives. Wish-lists are treatment site specific and are constructed in an iterative tuning process, together with the treating physician. Although clinically delivered manual plans serve as an initial reference for wish-list generation, the final goal is always to supersede the manual plan quality.

### Exploration of Patient-Specific Trade-Offs Between Target Coverage and Radiation-Induced Toxicity

In a recent study, Sharfo et al. (17) used automated treatment planning to investigate the quality of dose distributions delivered in the HYPRO trial (19). To that purpose, logistic regression analyses was used to develop an NTCP model (Equation 1) for grade ≥ 2 GI toxicity, based on scored toxicities, delivered doses and non-dosimetric predictive parameters.

(1) $$\begin{array}{l} NTCP=\frac{1}{1+e^{-6.362+B\cdot 2.083+D\cdot 0.608+T\cdot 0.406+E\cdot 0.084}} \end{array}$$

B = Baseline GI toxicity (yes/no), D = Diabetes (yes/no), T = High risk treatment group (yes/no) (19), and E = rectum gEUD_EQD2Gy_(7.7).

Here we used this model to systematically investigate patient-specific trade-offs between predicted GI toxicity and PTV_Low_ coverage. Seven plans were generated for each patient to quantify risk reductions associated with reductions in coverage from the clinical ≥99% to as low as 90% for otherwise highly similar dose distributions.

A sub-group of the patients in the study cohort had diabetes as a co-morbidity. However, to systematically explore diabetes as a co-morbidity, analyses were performed both assuming that all patients had diabetes or none of them had.

### Generated Treatment Plans

Erasmus-iCycle was used to automatically generate VMAT plans with 10 MV photon beams. Starting point for the plan generations was a slightly modified version of the wish-list developed by Sharfo et al. (17) for automated generation of plans with ≥99% coverage for both PTVs, in line with the HYPRO protocol. In this study, this wish-list was used to generate for each patient the so-called ‘clinical plan’ which is a high-quality Pareto-optimal plan with the currently required ≥99% coverage for both PTVs. (Note: these are not the clinically delivered plans, which were manually generated and of lower quality (17). The six alternative plans with various PTV_Low_ coverages in the range 99%−90% were generated with modified versions of this wish-list (as specified in section Wish-Lists) aiming for increased rectum sparing while guaranteeing high similarity with the clinical plan for other dose parameters.

### Wish-Lists

The applied wish-lists are described in Table 1 with some explanations in the following text. In Erasmus-iCycle, target coverage is generally optimized by minimizing a logarithmic tumor control probability (LTCP) cost function (Equation 2) (25),

(2) $$\begin{array}{l} LTCP=\frac{1}{m}\sum_{j=1}^{m}e^{(-\alpha \left(d_{j}-PD\right)} \end{array}$$

where m is the number of voxels in the target, PD the prescribed dose, d_j_ the dose in voxel j, and α the cell sensitivity parameter (26). A ≥99% coverage for PTV_High_ was for all generated plans achieved using a goal value of 0.8. Minimum dose constraints for CTV_High_ and CTV_Low_ guaranteed that CTV coverage was always maintained when reducing PTV_Low_ coverage.

**Table 1:** Wish-lists used for automated plan generations in this study.

**CONSTRAINTS**			
	**Structure**	**Constraint function**	**Limit**
	PTV_High_	Maximum dose	<105% of PD_High_
	PTV_High_	Mean dose	<100.5% of PD_High_
	PTV_Low_-(PTV_High_ exp by 2.5mm)	Maximum dose	<95% of PD_High_
	PTV Shell 50	Maximum dose	<50% of PD_High_
	Rectum	Maximum dose	<102% of PD_High_
	Anus	Maximum dose	<102% of PD_High_
	Patient	Maximum dose	<105% of PD_High_
	CTV_High_	Minimum dose	>95% of PD_High_
	CTV_Low_	Minimum dose	>95% of PD_Low_
**OBJECTIVES**			
**Priority**	**Structure**	**Aim & objective function**	**Goal value (Sufficient)**
1	PTV_High_	↓ LTCP(99.5% of PD_High_,α=0.8)	0.8 (0.8)
***2***	***PTV**_***Low***_**-RectumPRV***	**↓*****LTCP(PD**_***Low***_,***α*****=1.4)***	***0.4 (0.4)***
***3***	***PTV**_***Low***_*	**↓*****LTCP(PD**_***Low***_,***α*****=1.4)***	***X (X)***
4	Rectum	↓ gEUD(7.7)	0
5	Entrance Dose	↓ Maximum dose	<20% PD_Low_
5	PTV Shell 5	↓ Maximum dose	<80% PD_Low_
6	Rectum	↓ Mean dose	5
7	Anus	↓ Mean dose	5
8	PTV Shell 15	↓ Maximum dose	<50% PD_Low_
8	PTV Shell 25	↓ Maximum dose	<30% PD_Low_
***9***	***Bladder***	**↓*****Mean dose***	***5***
10	Hip left	↓ Maximum dose	40
10	Hip right	↓ Maximum dose	40

*Bold/italic lines are different for the clinical plans and alternative plans (see text). Minimum values to CTVs were set 2 Gy higher to account for voxel sampling in the optimizations. PD_High_, prescribed dose for PTV_High_ (64.6 Gy); PD_Low_, prescribed dose for PTV_Low_ (57.76 Gy); gEUD(k), generalized equivalent uniform dose; k, volume parameter; LTCP(PD,α), logarithmic tumor control probability (25); with α, cell sensitivity; OAR, organ at risk; ↓, minimization; ↑, maximization*.

For generation of the clinical plan, the priority 2 cost function was disabled and a goal value of X = 0.4 was used in priority 3 to always acquire >99% coverage for PTV_Low_ (the LTCP cost function was applied to the entire PTV_Low_, including the overlapping area with the rectum). Rectum sparing was obtained by optimizing a gEUD(k) with k equal to 7.7, in line with the NTCP model (Equation 1). Conformality of the dose outside the PTVs was controlled by a set of maximum dose objectives (priorities 5 and 8), assigned to concentric shells around PTV_Low_.

For generation of the six plans with reduced PTV_Low_ coverage, modifications in the wish-list were made at the level of the bold/italic lines in Table 1. The aim was always to have PTV_Low_ underdosages in the most promising regions for GI NTCP reduction, i.e., where rectum was overlapping with the PTV_Low_ and its surroundings, without compromising the CTV doses and while keeping the remainder of the dose distribution as similar as possible to the clinical one. To this purpose, the priority 2 objective was introduced for dose optimization in the PTV_Low_-RectumPRV structure in which the overlapping rectum expanded by a margin was subtracted from the PTV_Low_. The applied PRV margins were 25, 20, 15, or 10 mm for patient-specific PTV_Low_ and rectum overlapping areas of <4, <6, <7, or >7%, respectively. An LTCP cost function with a goal value of 0.4 was used to always cover >99% of PTV_Low_-RectumPRV.

To obtain plans with various PTV_Low_ coverages <99%, the LTCP in priority 3 was now used for partial recoveries of the PTV_Low_ coverage in a controlled way. This was performed by using well-selected (patient-independent) X-values in priority 3 that were different for each of the six plans generated with reduced PTV_Low_ coverage. For generation of the plans with reduced PTV_Low_ coverage, the bladder D_Mean_ objective in priority 9 was removed, while a bladder D_Mean_ constraint was added with a limit value equal to the patient-specific bladder D_Mean_ obtained in the clinical plan. This was done in order to avoid dose being pushed away from the rectum toward the bladder.

Creation of the appropriate wish-lists was performed in a tuning process involving CT-scans of a set of 10 patients.

## Results

**Figure 1:**
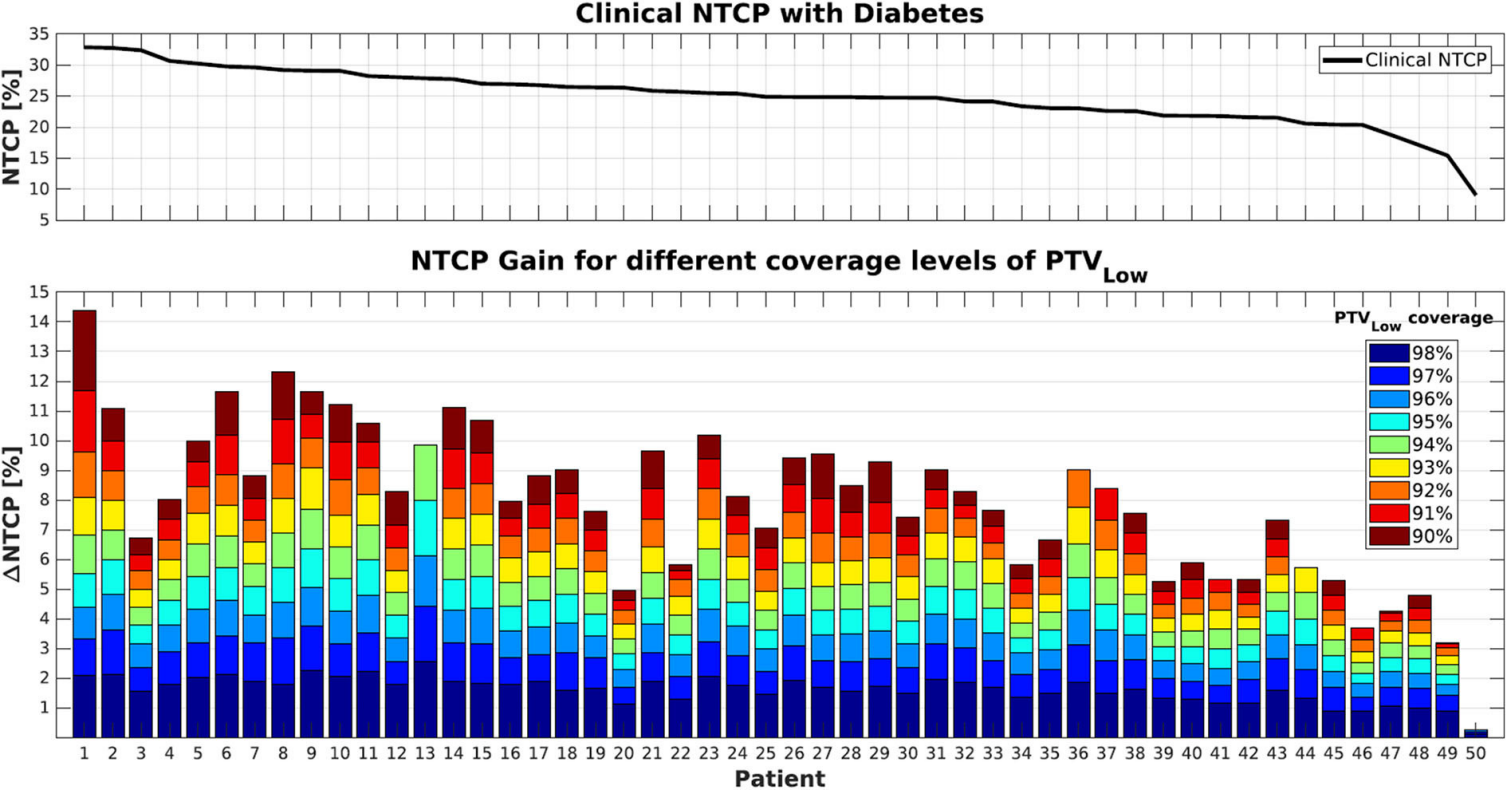
**(Top)** NTCP values for the clinical plans (PTV_Low_ coverage ≥99%) in case of diabetes as a co-morbidity. **(Bottom)** Cumulative NTCP reductions for decreasing levels of PTV_Low_ coverage. Patients were sorted according to their clinical NTCP as visualized in the top panel.

**Figure 2:**
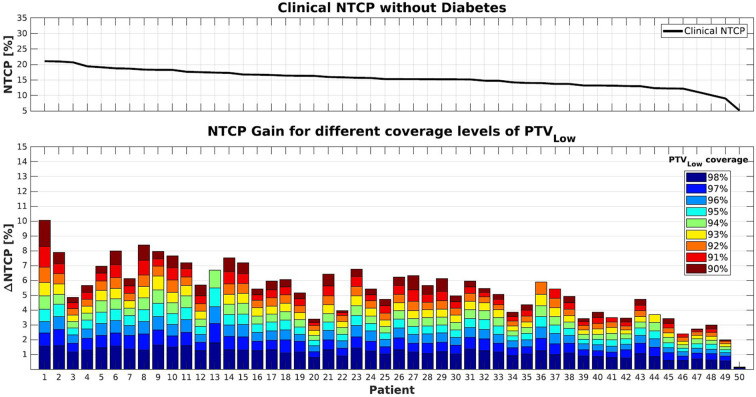
**(Top)** NTCP values for the clinical plans (PTV_Low_ coverage ≥99%). **(Bottom)** Cumulative NTCP reductions for decreasing levels of PTV_Low_ coverage. Patients were supposed not to have diabetes. Patient sorting along the x-axis was the same as for Figure 1.

Figures 1, 2 show NTCP reductions for the 50 study patients as a function of the loss in PTV_Low_ dose coverage. Figure 1 is valid in case of diabetes, while for Figure 2 we assumed that there was no diabetes. As explained in the M&M section, reductions in PTV_Low_ coverage in the six alternative plans for each patient were obtained with (convex) LTCP cost functions. Convexity avoids getting trapped in local minima, but with the LTCP cost function, obtained PTV_Low_ coverage values vary somewhat between patients. For generation of Figures 1, 2, NTCPs for the defined coverage reductions were for each patient obtained by piecewise linear interpolations between the generated plans. The different colors show the impact of incremental underdosage steps of 1% in PTV_Low_ on obtained NTCP. For some patients (e.g., patient 13), reducing the coverage to as low as 90% was not possible, possibly due to not sufficiently large PRV margins or conflicting constraints on the PTV_High_ and the CTVs dose requirements. For patient 50, accepting lower PTV_Low_ coverage did not result in any NTCP reduction because of lack in overlap between PTV_Low_ and rectum (see also Figure 6).

Following Equation 1, NTCP values were indeed higher in case patients had diabetes (compare upper panels of Figures 1, 2). On the other hand, NTCP reductions were also larger in case of diabetes. For a PTV_Low_ coverage of 95%, average NTCP reductions of 4.3% (0.3–8.0%) and 2.9% (0.2–5.5%) were obtained with or without diabetes, respectively. For 90% coverage, the obtained NTCP reductions increased to 8.3% (0.3–14.4%) and 5.6% (2.0–10.1%), respectively. Both with and without diabetes, there was an overall trend toward enhanced NTCP reductions for patients with the highest clinical NTCPs (lower panels Figures 1, 2). On the other hand, large inter-patient variations were observed. For example, patients 1 and 3 had similar clinical NTCPs, but a large difference in achievable NTCP reductions. Moreover, similar NTCP reductions were observed for different costs in PTV_Low_ coverage. For example, patients 12 and 14 have similar NTCP reductions of ~10% accepting 94% or 91% PTV_Low_ coverage instead of 99% (Figure 1). Observed maximum NTCP reductions ranged from > 14% (patient 1) to <1% for patient 50, depending on differences in anatomy (see Figure 6).

**Figure 3:**
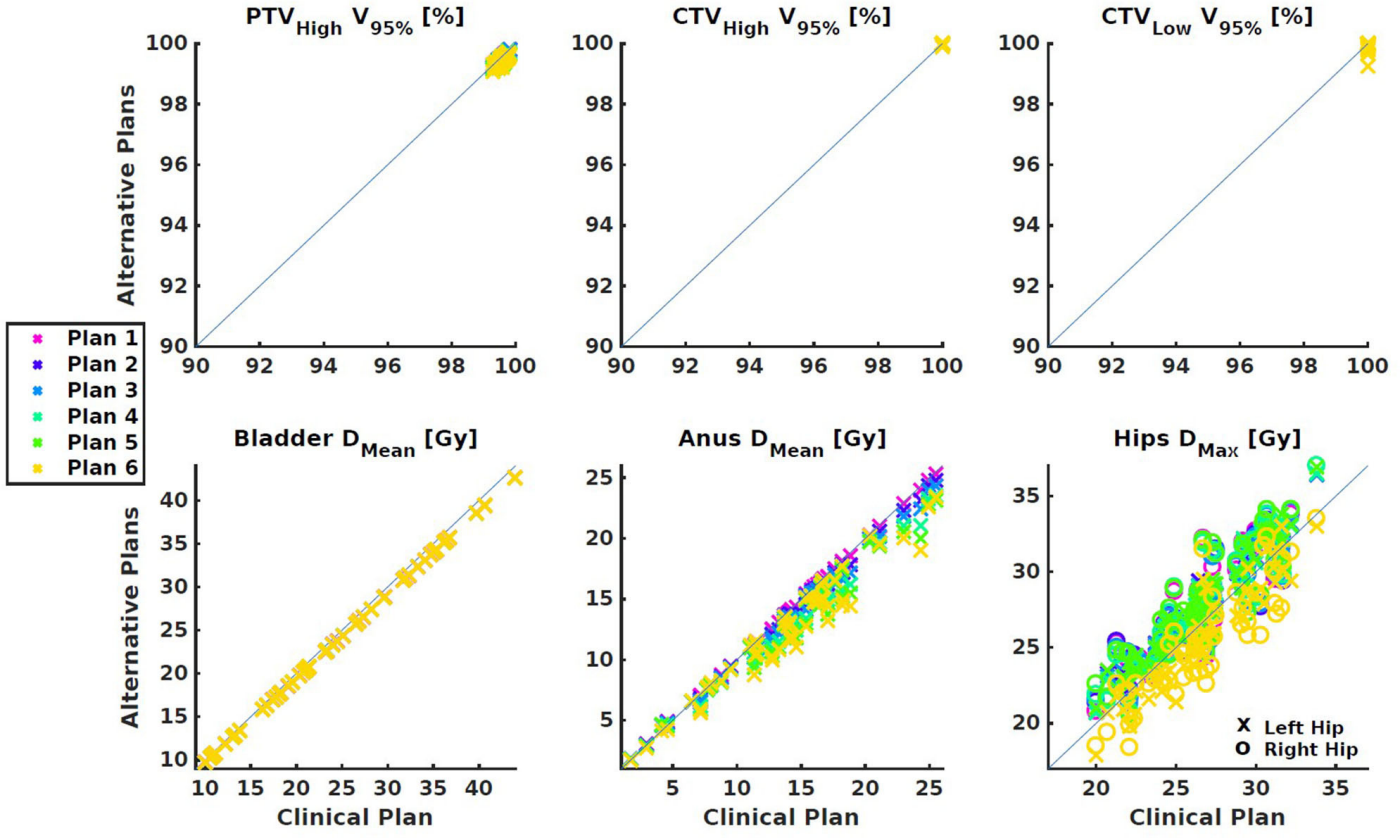
Dose parameter differences between the clinical plan and the alternative plans with reduced PTV_Low_ coverages. **(Top)** Targets. **(Bottom)** OARs. All differences were small and within clinically acceptable limits.

**Figure 4:**
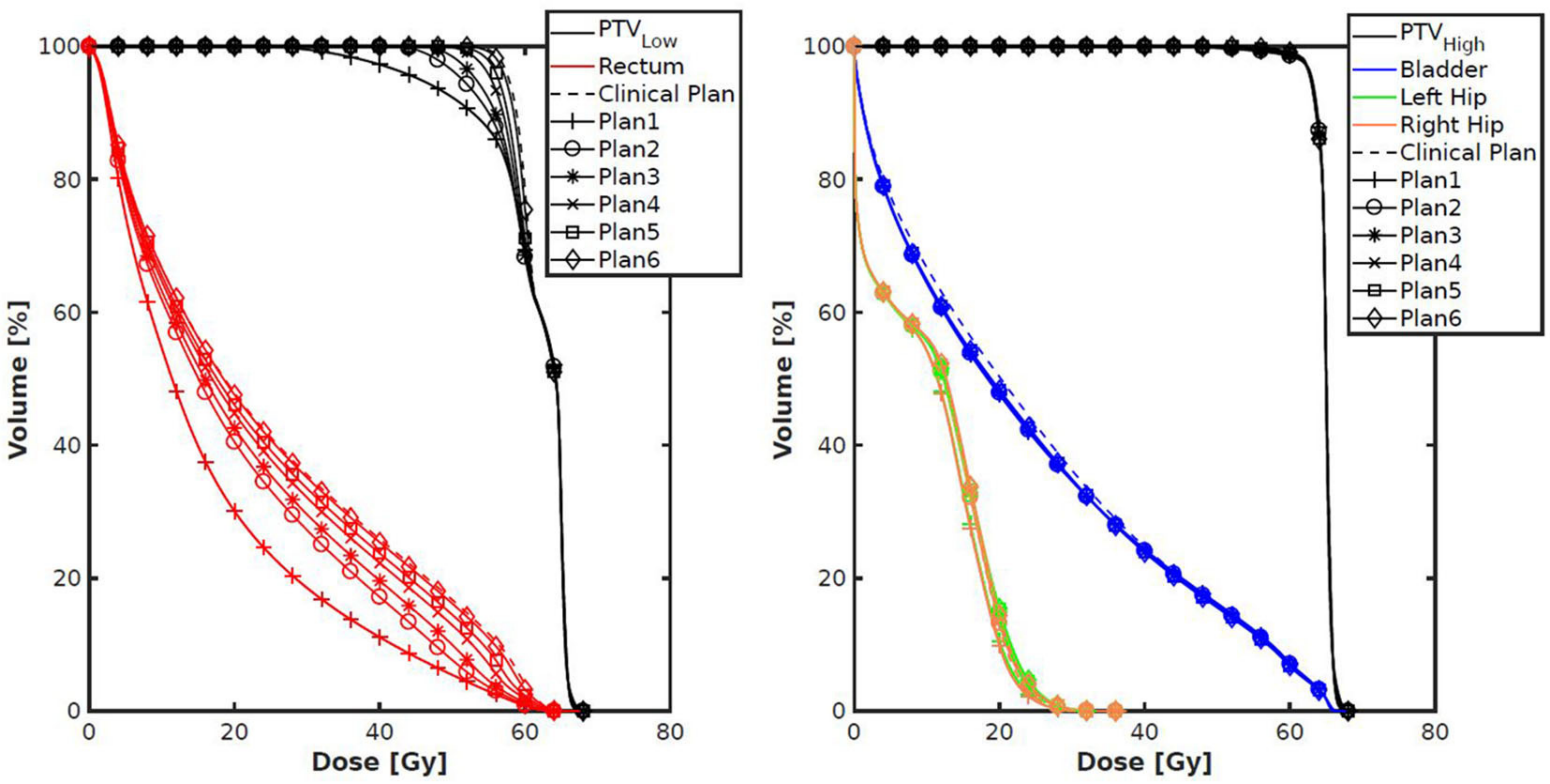
Population average DVHs for each of the seven plans generated per patient. The left panel shows a clear (intended) trade-off between rectum dose and coverage of PTV_Low_. The right panel shows very small differences for other structures.

**Figure 5:**
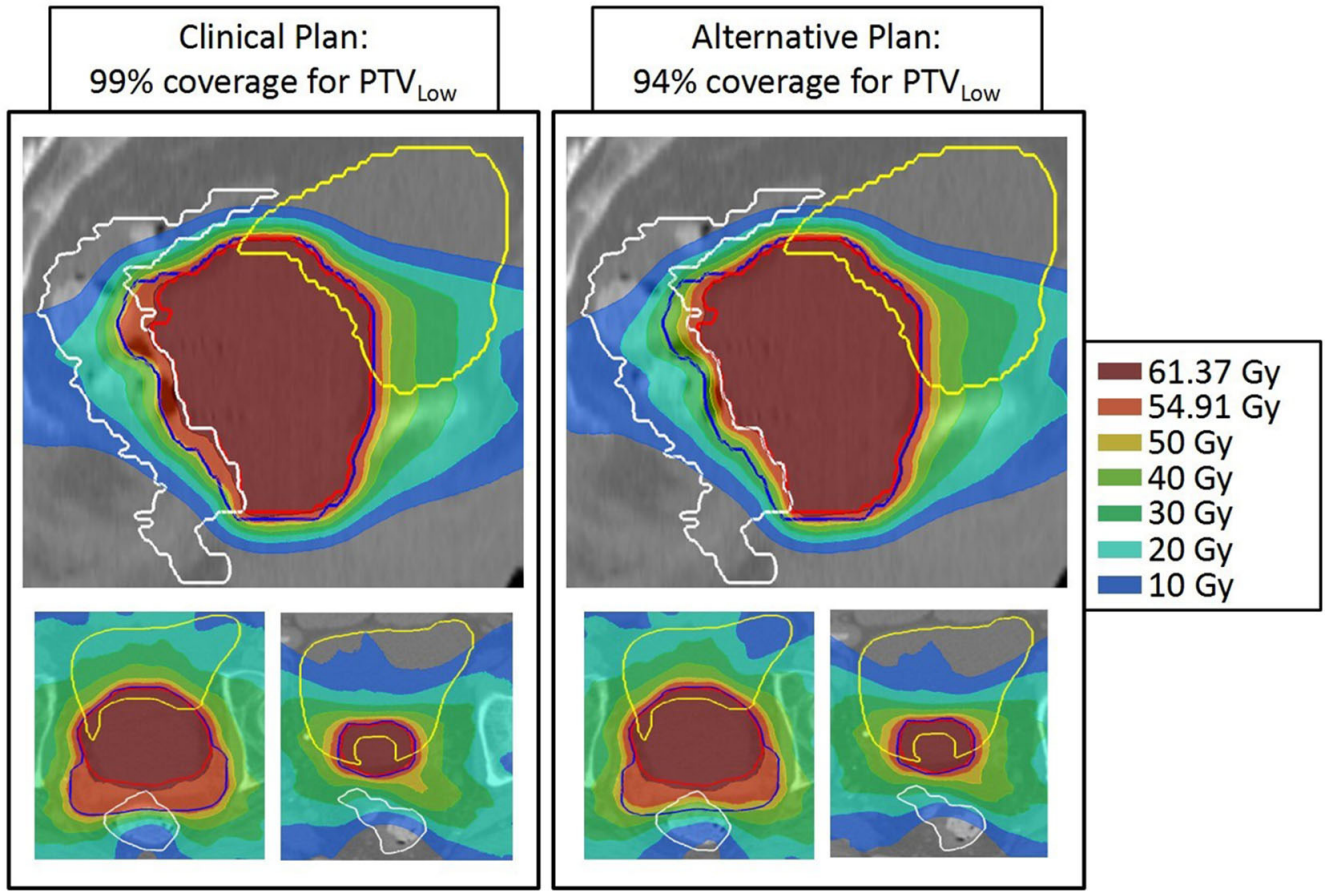
Dose distributions of patient 13 for 99% (left) and 94% PTV_Low_ dose coverage. For both patients, **top**: sagittal view through isoc., **bottom**: axial views at two levels. Structures: red = PTV_High_, blue = PTV_Low_, white = rectum, and yellow = bladder. Apart from the dose in the posterior part of PTV_Low_, dose distributions are highly similar.

Figures 3, 4 show the differences between clinical and alternative plans on a per patient base and in population DVHs, respectively. They demonstrate that the enforced PTV_Low_ coverage reductions mainly had an impact on rectum sparing while having a clinically insignificant dosimetric impact on PTV_High_, CTV_High_, CTV_Low_, bladder, anus and hips, as intended (section Generated Treatment Plans). Figure 5 shows for an example patient highly similar dose distributions, except for the region of overlap between rectum and PTV_Low_.

**Figure 6:**
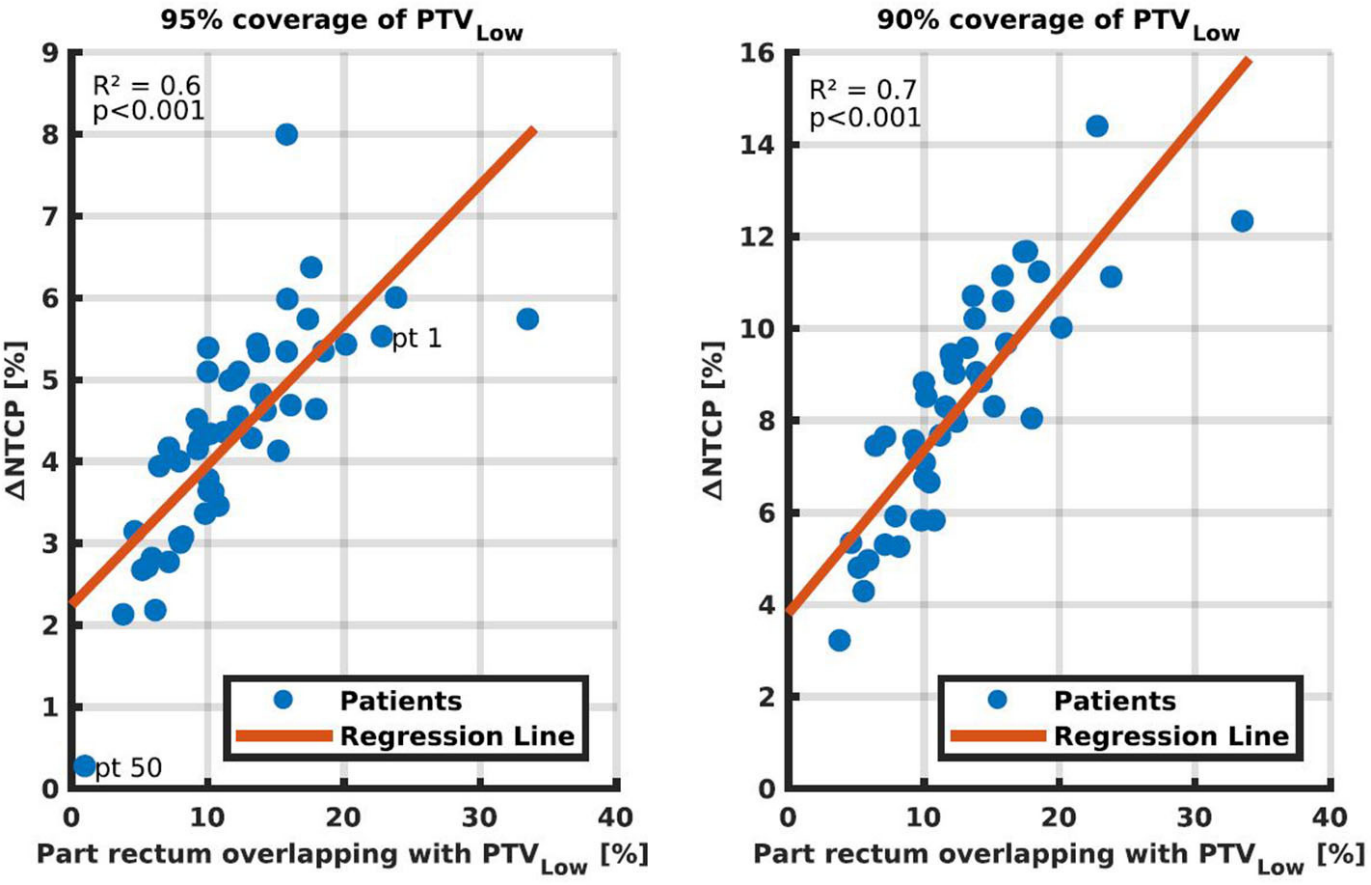
NTCP reductions by going from ≥99% coverage for PTV_Low_ to 95% **(Left)** and 95% **(Right)**, as a function of the percentage of rectum overlapping with PTV_Low_. Each dot represents one of the fifty study patients. In the left panel, patients 1 and 50 are marked for discussions in the text.

In Figure 6 we investigated the extent of feasible NTCP reduction as a function of overlap between rectum and PTV_Low_. Although, there is an overall trend toward more reduction with larger overlap, there are inter-patient variations with *R*^2^ equal to 0.6 and 0.7, for 95 and 90% PTV_Low_ coverage, respectively.

**Figure 7:**
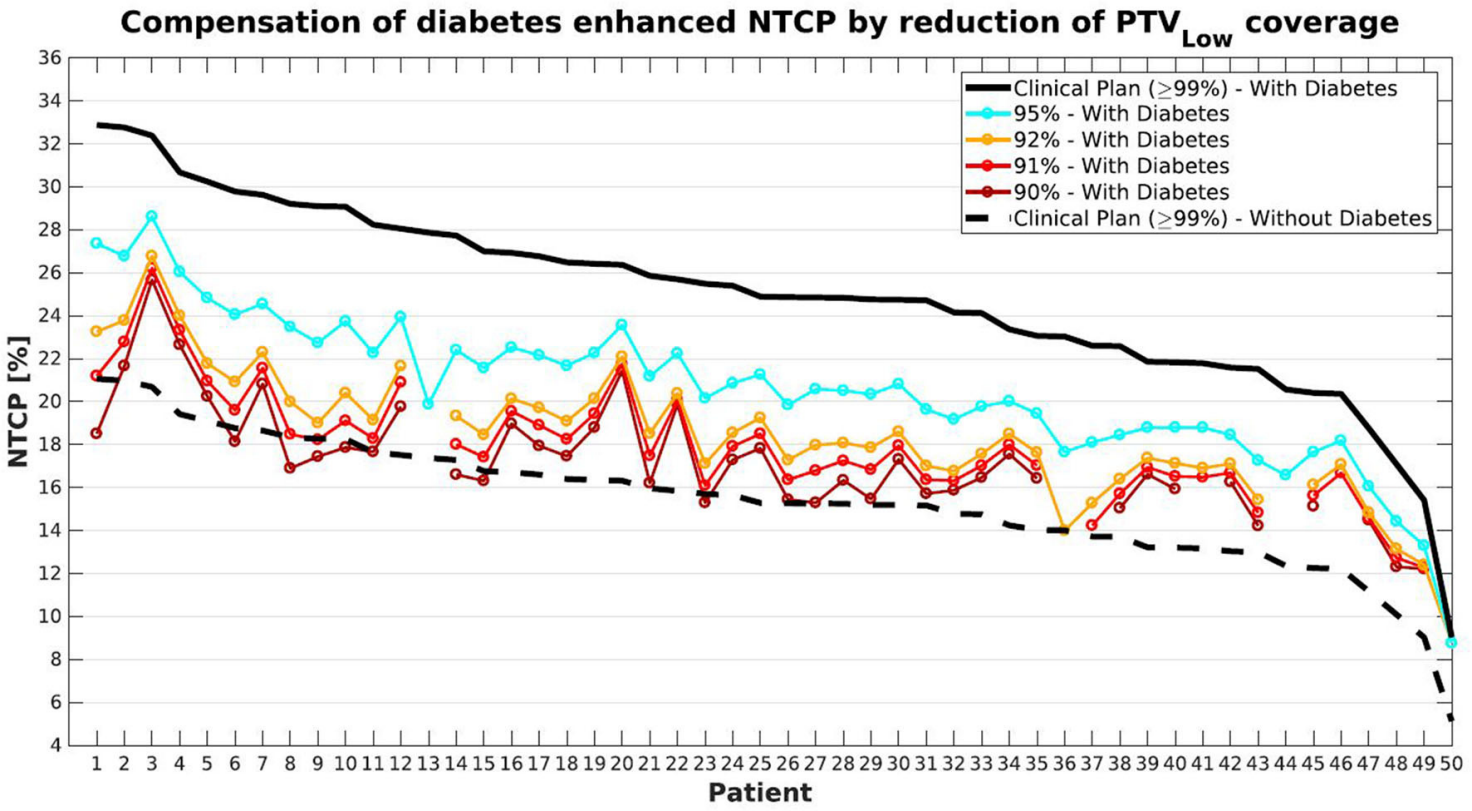
Compensation for diabetes induced-enhancement of predicted NTCPs (compare black solid and dashed lines) by reducing PTV_Low_ coverage levels. With gradual decreases in coverage, NTCPs with diabetes gradually approach the dashed curve for NTCPs without diabetes. Patient sorting along the x-axis was the same as for Figure 1.

For the 50 patients in this study, presence of diabetes resulted in an average increase in clinical NTCP from 15.4 ± 3.0% (1SD) to 24.9 ± 4.5% (1SD) (compare also the upper panels of Figures 1, 2). Figure 7 explores opportunities for mitigation of enhanced toxicity risk due to diabetes by reducing required PTV_Low_ coverage. Clearly, depending on the allowed coverage reduction and the patient anatomy, NTCP enhancements due to diabetes could be largely compensated. For some patients, (e.g., 1, 6, and 8) the impact of diabetes could be completely canceled when using a coverage of 90–91%. Other patients (e.g., 3 and 20) demonstrate quite large residual differences in NTCP with and without diabetes, for reduced PTV_Low_ coverages.

## Discussion

In this study, we have used prostate cancer radiotherapy as a model for development of a clinically feasible workflow for application of automated planning for assessment of patient-specific trade-offs between treatment goals. All plans were generated fully automatically, i.e., without any manual fine-tuning. With carefully designed, patient-independent variations in the autoplanning configuration (i.e., wish-list), the PTV_Low_ coverage could be varied in a controlled way in the range 99–90% to reduce the predicted NTCP, without significant further changes in the dose distributions. In particular, CTV coverage remained 100%, PTV_High_ coverage was kept at ≥99%, and bladder dose did also not significantly change. For each patient, the obtained bladder D_Mean_ in the clinical plan (PTV_Low_ coverage ≥99%) was used as constraint in the generation of the six other plans with reduced PTV_Low_ coverage. It was demonstrated that large, but highly patient-specific NTCP reductions could be obtained. For a PTV_Low_ coverage of 90%, observed NTCP reductions ranged from 14.4 to 0.1%, compared to 99% coverage, depending on the patient anatomy. Reductions in required PTV_Low_ coverage could to a large extent make up for diabetes as a co-morbidity, again depending on patient anatomy. To the best of our knowledge, this is the first study that proposes the use of automated planning for patient-specific exploration of opportunities for dosimetric compensation of non-dosimetric toxicity risk factors.

Automated treatment plan generation required about 1–2 h per treatment plan. No manual interaction was required at any step of the procedure. Therefore, multiple plans could be run, sequentially or in parallel, over the night. Generation of a wish-list generally takes several weeks. This is a one-time effort and should be seen as an upfront time-investment, which saves a lot of manual planning time at a later stage. Specifically for this project, the wish-list was already developed in a previous study (17).

Observed NTCP reductions correlated to some extent with the volume of rectum overlapping with PTV_Low_ (Figure 6, *R*^2^ = 0.6–07). Once a correlation model is built based on the plans generated with the proposed method, the regression lines might be of use as a tool for selection of the PTV_Low_ coverage region of interest, or for selection of patients. That is, the proposed method could be applied only to patients and/or to PTV_Low_ levels that show to be more promising in NTCP reduction. However, even in the relatively easy treatment site of prostate cancer, a not too strong correlation was found. Different parameters may be investigated, but for more challenging treatment sites finding predictors for NTCP reduction may be even more complex. The presented method, on the other hand, only requires computation time once the procedure is defined.

In the wish-lists applied in this study, concentric shells at distances of 5, 15, 25, and 50 mm from the PTV edge were used to control plan conformality (Table 1). The limit and goal values were the same for all patients and all plans. Initially, we did however try to get further NTCP reductions by loosening conformality goal values. This was not successful; conformality worsened but NTCPs remained practically unchanged.

Equation 1 was used for NTCP prediction in this study, as our patients were treated in the context of the HYPRO trial, and Equation 1 was derived for these patients. Important to note is that various alternative predictive models exist (11, 15), which could possibly have resulted in different conclusions, or could have resulted in different approaches for lowering NTCPs. Direct use of Equation 1 in this study was limited to plan evaluations, i.e., Equation 1 was not used in the wish-list for plan generations (see Table 1). For planning, we generally prefer to use convex cost functions to avoid getting trapped in local minima, and the NTCP expression in Equation 1 is not convex. Alternatively, the (convex) rectum gEUD (7.7), as used in Equation 1, was directly applied as an objective function (priority 4 in Table 1).

The proposed method to explore trade-offs in planning goals has some similarities with the well-known Pareto navigation, using a graphical user interface with sliders to find a clinically favorable plan (8, 26–31). Also in that method, multiple plans are automatically generated for manual plan selection. There are, however, important differences. The most important difference is that for each patient, we first generate a high-quality, Pareto optimal plan (“wish-point”) with clinically most desired PTV_Low_ coverage (≥99%). For each patient, this plan is then used as anchor point for patient-specific generation of the plans with slightly reduced PTV_Low_ coverage, using the bladder dose obtained in the wish-point plan as constraint. In the proposed workflow, only plans are generated that are useful for the desired analyses. In conventional generation of plans for Pareto navigation, there is no knowledge of the “wish-point,” and generation of plans is less focused. Due to our highly focused plan generation, only few plans are needed for the analyses. In this study we used seven plans per patient. This number was not optimized in terms of finding the minimum number of required plans. The aim was to include for all patients, the full range of PTV_Low_ coverages from 99 to 90%. If a clinical protocol has more precise directions for reductions in PTV_Low_ coverage, for sure even fewer plans need to be generated.

PTV margins are generally used to minimize risk in CTV miss. In this paper, we kept all margins unchanged, but allowed doses in the overlap area of PTV_Low_ with rectum to get lower than in the clinical plan. Coverages in PTV_High_ and CTV were always maintained. With this approach, the risk of CTV miss was minimized, but still (at least potentially) enhanced compared to regular clinical planning. Therefore, clinical introduction of this type of workflow is not trivial. Extensive computer simulations could be performed to assess the true risks, taking into account the clinically applied image-guided approach. Clinical introduction could well be performed in a formal study. Anyway, it seems that patient selection could be important, with patients with a high clinical NTCP (e.g., related to an unfavorable anatomy or diabetes) and a large potential for NTCP reductions, as best candidates. It is important to realize that we used in this study our clinically required PTV coverage level of 99%. In many studies, coverages of 95% were reported (32).

We have investigated trade-offs between PTV coverage and GI NTCP for prostate cancer but believe that the proposed methodology could also be applied for other tumor sites. The system could also be used to explore patient-specific trade-offs between various toxicities for fixed PTV coverage. Focusing on balances between toxicities instead of toxicity vs. PTV coverage could ease clinical implementation. The developed workflow could potentially also be used in shared decision making studies.

## Conclusion

A novel, clinically feasible workflow has been proposed for the use of automated planning to systematically explore patient-specific trade-offs between various treatment aims. For prostate cancer, the patient-specific balance between PTV coverage and predicted GI toxicity risk was explored. Opportunities for compensating significantly enhanced predicted toxicity risk related to diabetes by reducing the PTV coverage were investigated as well. Large variations in potential benefit were observed in the fifty study patients. The proposed system could play an important role in further high-precision personalization of patient care.

## Data Availability Statement

The datasets generated for this study are available on request to the corresponding author.

## Ethics Statement

Ethical review and approval was not required for the study on human participants in accordance with the local legislation and institutional requirements. Written informed consent for participation was not required for this study in accordance with the national legislation and the institutional requirements.

## Author Contributions

RB: conducting main research and writing manuscript. LR and BH: supervision of research and writing manuscript. AS: brainstorming about research and writing manuscript. SB: developing code and writing manuscript. WH: data collection and writing manuscript. LI: PI of Data collection and writing manuscript. All authors contributed to the article and approved the submitted version.

## Conflict of Interest

The authors declare that the research was conducted in the absence of any commercial or financial relationships that could be construed as a potential conflict of interest.
